# Time to local recurrence as a predictor of survival in unrecetable gastric cancer patients after radical gastrectomy

**DOI:** 10.18632/oncotarget.19038

**Published:** 2017-07-06

**Authors:** Hui Li, Xiangyu Jin, Peng Liu, Wei Hong

**Affiliations:** ^1^ Department of Chemotherapy, Zhejiang Cancer Hospital, Hangzhou, Zhejiang, China; ^2^ Department of Radiotherapy, Zhejiang Cancer Hospital, Hangzhou, Zhejiang, China

**Keywords:** gastric cancer, local recurrence, time to local recurrence, survival, prognostic factors

## Abstract

Local recurrence is common after radical surgery. However, the factors that contribute to survival after local recurrence remain unclear. In this retrospective study we analyzed the relationship between time to recurrence and survival after recurrence in 74 patients with locally recurrent gastric cancer. All patients received palliative radiotherapy with or without chemotherapy. The patients were divided into two groups according to the time between gastrectomy and local recurrence: early local recurrence (ELR, < 12 months after primary surgery), and late local recurrence (LLR, ≥12 months after primary surgery). The median overall survival (OS) time was 15 months for patients with ELR and 25 months for LLR patients. Univariate and multivariate analysis revealed that time to local recurrence was significantly associated with OS after local recurrence (*P* = 0.001). The hazard ratio of ELR compared with LLR patients was 0.442 (95% confidence interval: 0.264-0.741). These results indicate that early local recurrence predicted poor prognosis in gastric cancer patients with unresectable local recurrence.

## INTRODUCTION

Gastric cancer is the second leading cause of cancer-related mortality, and remains the fourth most common cancer worldwide [1]. In China, the morbidity and mortality of gastric cancer are higher than all but one other type of cancers [2].

Several studies of clinical follow-up, reoperation and autopsies have reported that 40%-60% of patients with completely resected gstage II or III gastric cancer developed a locoregional recurrence before adjuvant therapy was applied [3]. Local-regional failure most often occurs in anastomosis, followed by the stomach bed and undissected regional nodes [4]. Due to dysphagia, bleeding and pain, some patients with locoregional recurrence cannot be treated surgically and thus receive only palliative therapy [5]. The median overall survival (OS) of these patients was only 5 months (range 0.2-82 months) [6].

Owing to the increased diagnosis of earlier stage disease, better preoperative staging and improved adjuvant treatment strategies, the rate of locoregional recurrence and long-term survival have been greatly improved. The INT0116 and RTOG9904 trials evaluated the outcomes of adjuvant therapy for patients with gastric cancer at high risk for recurrence [7]. These studies reported that application of preoperative and postoperative chemoradiotherapy (CRT) achieved good outcomes in patients with gastric cancer who received curative resection. Thus, these therapies were recommended by the 2013 National Comprehensive Cancer Network (NCCN) guidelines. Cunningham *et al.* reported that perioperative chemotherapy is beneficial for patients with resectable stage II/III gastric cancer [8]. Sakuramoto *et al.* compared the efficacy of surgery plus postoperative S1 adjuvant chemotherapy with surgery alone in patients with gastric cancer. Thus, radiotherapy has been excluded from standard regimens [9].

A number of factors have been reported to be associated with locoregional recurrence [10]. However, to our knowledge, significant predictors of survival time after local-regional recurrence have not been reported. Patients with late recurrence are reported to have a better prognosis than those with early recurrence in rectal cancer [11]. Therefore, the aim of this study was to investigate whether the time from primary surgery to detection of a locoregional recurrence is a significant predictor of OS after local recurrence of gastric cancer.

## RESULTS

### Baseline patient characteristics

The medical records of 74 patients with locoregional recurrence after complete gastrectomy were retrospectively reviewed, including 52 male patients and 22 female patients, aged between 28 and 80 years (median: 53.5 years). The baseline characteristics of these patients’ were recorded at the point when their primary tumors were detected (Table 1). The primary tumors were categorized as T1 (2, 2.7%), T2 (9, 12.2%), T3 (15, 20.3%), T4 (48, 64.8%). These patients’ characteristics were again recorded when their recurrent tumors were detected (Table 2). According to the AJCC TNM staging system the regional lymph node metastases were categorized as N0 (6, 8.1%), N1 (11, 14.9%), N2 (1, 23.0%) and N3 (40, 54.1%). Vascular invasion was detected in 20 (27.0%) patients. Fifteen (20.3%) patients had signet-ring cell carcinoma, 44 (59.5%) had poorly-differentiated adenocarcinoma, 9 (12.2%) had moderately-differentiated adenocarcinoma and 6 (8.1%) had highly-differentiated adenocarcinoma. The median age of these patients at recurrence was 55 years (range: 28-81 years). The diameter of recurrent lesions ranged from 0.5 to 6.7 cm (mean: 2.53 cm). The recurrence sites were anastomotic or remnant recurrence in 19 patients (25.7%) and local lymph nodes in 55 (74.3%). In ELR group, only one patient synchronously had anastomotic and lymph node recurrence. And three patients in LLR group had both anastomotic and lymph node recurrence and 3 patients in LLR group.

**Table 1:** Clinicopathological characteristics of patients with early and late locoregional recurrence at time of primary tumor detection

	ELR(<12m) (N=30)	LLR(≥12m) (N=44)	P
**Age**			
<65	28(40.6%)	41(59.4%)	
≥65	2(40.0%)	3(60.0%)	0.980
**Gender**			
Male	20(38.5%)	32(61.5%)	
Female	10(45.5%)	12(54.5%)	0.575
**T stage**			
T1-T2	4(33.3%)	8(66.7%)	
T3-T4	26(41.9%)	36(58.1%)	0.579
**N stage**			
N0-N1	7(41.2%)	10(58.8%)	
N2-N3	23(40.4%)	34(59.6%)	0.951
**Vascular invasion**			
NO	19(35.2%)	35(64.8%)	
YES	11(55.0%)	9(45.0%)	0.123
**Pathological type**			
SRC	5(33.3%)	10(66.7%)	
PDA	20(45.5%)	24(54.5%)	
MDA	4 (44.4%)	5(55.6%)	
HDA	1 (16.7%)	5(83.3%)	0.524

SRC: signet-Ring Cell carcinoma

PDA: poor-differentiated adenocarcinom

MDA: middler-differentiated adenocarcinom

HAD: high-differentiated adenocarcinom

**Table 2:** Patients’ clinicopathological characteristics at time of early or late locoregional recurrence

	ELR(<12m) (N=30)	LLR(≥12m) (N=44)	P
**Age**			
<65	22(43.1%)	29(56.9%)	
≥65	8(34.8%)	15(65.2%)	0.498
**Gender**			
Male	20(38.5%)	32(61.5%)	
Female	10(45.5%)	12(54.5%)	0.575
**Diameter of recurrent lesion**			
<2	15(46.9%)	17(53.1%)	
≥2	15(35.7%)	27(64.3%)	0.333
**Site of recurrence**			
Anastomotic recurrence	5(26.3%)	14(73.7%)	
Local lymph node recurrence	25(45.5%)	30(54.55)	0.143
**Response**			
CR+PR	4(19.0%)	17(81.0%)	
SD+PD	26(49.1%)	27(50.9%)	0.018
**Serum CA 72-4**			
≤6.9U/mL	17(32.1%)	36(67.9%)	
>6.9U/mL)	8(57.1%)	6(42.9%)	0.085
**Serum AFP**			
≤10ng/ml	24(37.5%)	40(62.5%)	
>10ng/ml	4(80.0%)	1(20.0%)	0.150
**Serum CEA**			
≤5ng/ml	17(34.7%)	32(65.3%)	
>5ng/ml	13(52.0%)	12(48.0%)	0.211
**Serum CA199**			
≤37U/mL	24(45.3%)	29(54.7%)	
>37U/mL	6(28.6%)	15(71.4%)	0.187

Patients were subjected to the therapy as described in the Materials and Methods. Six patients (8.1%) achieved a complete response, and the median OS time after recurrence was 36.5 months. Partial response was observed in 15 patients (20.3%) with a median OS after recurrence of 22 months. Forty-six patients (62.2%) had stable disease (SD), and seven patients (9.4%) had progressive disease (PD). The median OS time after recurrence of SD and PD was 18.5 and 9 months, respectively.

### Time to recurrence and clinicopathological characteristics of patients with primary tumors and recurrent tumors

In this study, local recurrence was detected within 12 months of primary gastrectomy in 30 patients (40.5%) (early local recurrence, ELR), and after 12 months in 44 patients (59.5%) (late local recurrence, LLR). The median time to recurrence was 14 months (range: 1-146 m). Time to recurrence was not significantly associated with patient age (*P* = 0.98)., sex (*P* = 0.575), T stage (*P* = 0.579), N stage (*P* = 0.951), vascular invasion (*P* = 0.123) or pathological type (*P* = 0.524). And no significant association was observed between diameters of recurrent lesion, site of recurrence and the time to recurrence. As for treatment response, the objective response rate (ORR), calculated as CR+PR in LLR patients was higher than the ELR patients (*P* = 0.018). Significantly more patients in the LLR group achieved CR or PR (26, 49.1%) than the ELR group (4, 19.0%). Fraction of patients with SD and PD in the LLR group was similar to that in the ELR group. The levels of tumor biomarkers including CA72-4 (*P* = 0.085), AFP (*P* = 0.150), CEA (*P* = 0.211) and CA199 (*P* = 0.187) at time of recurrence were not significantly associated with time to recurrence.

### Univariable and multivariable analysis of prognostic relevance of time to recurrence and clinicopathological parameters

**Figure 1:**
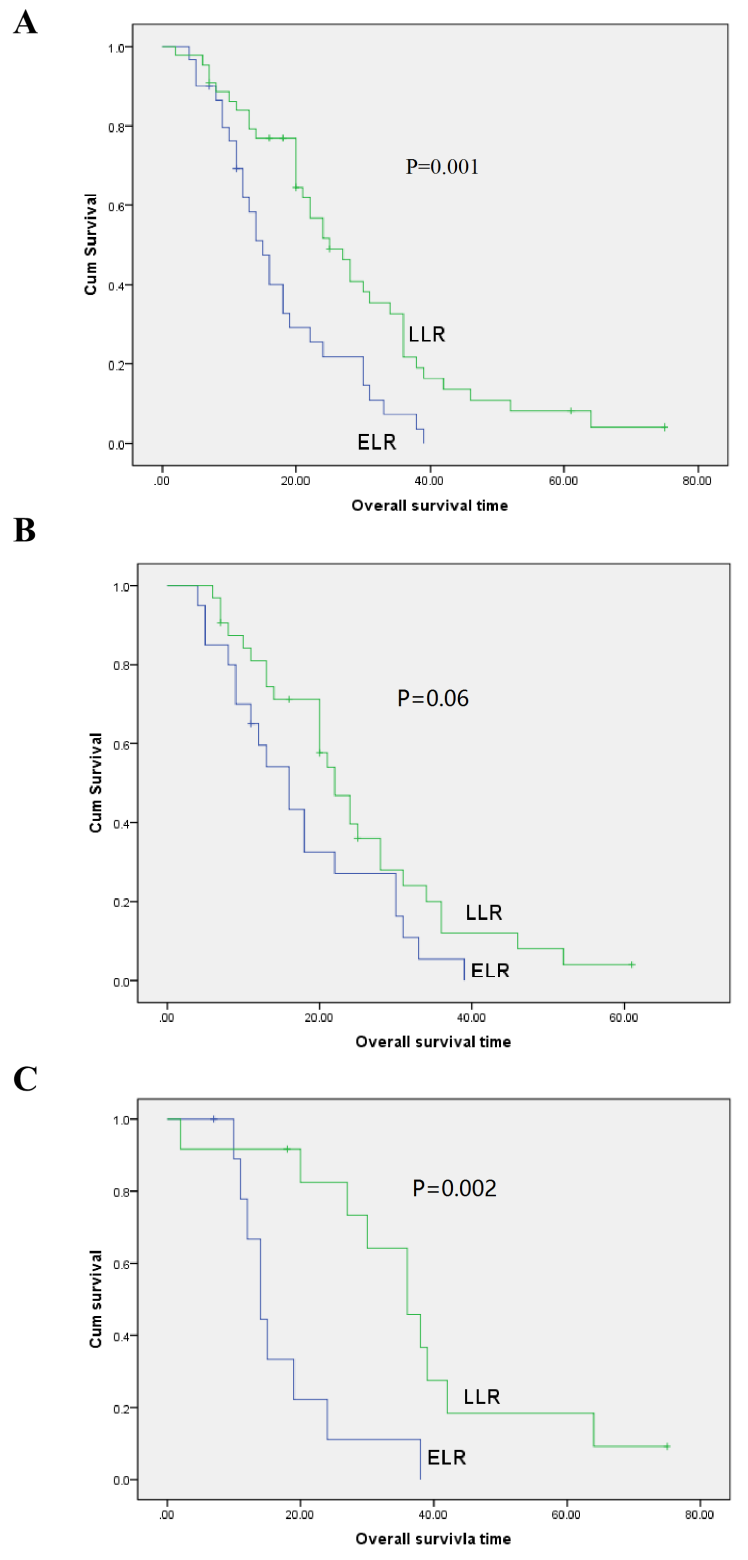
Overall survival curves with time to recurrence for gastric cancer patients with local recurrence in all patients (A., *P* = 0.001), male patients (B., *P* = 0.06), and female patients (C., *P* = 0.002)

**Figure 2:**
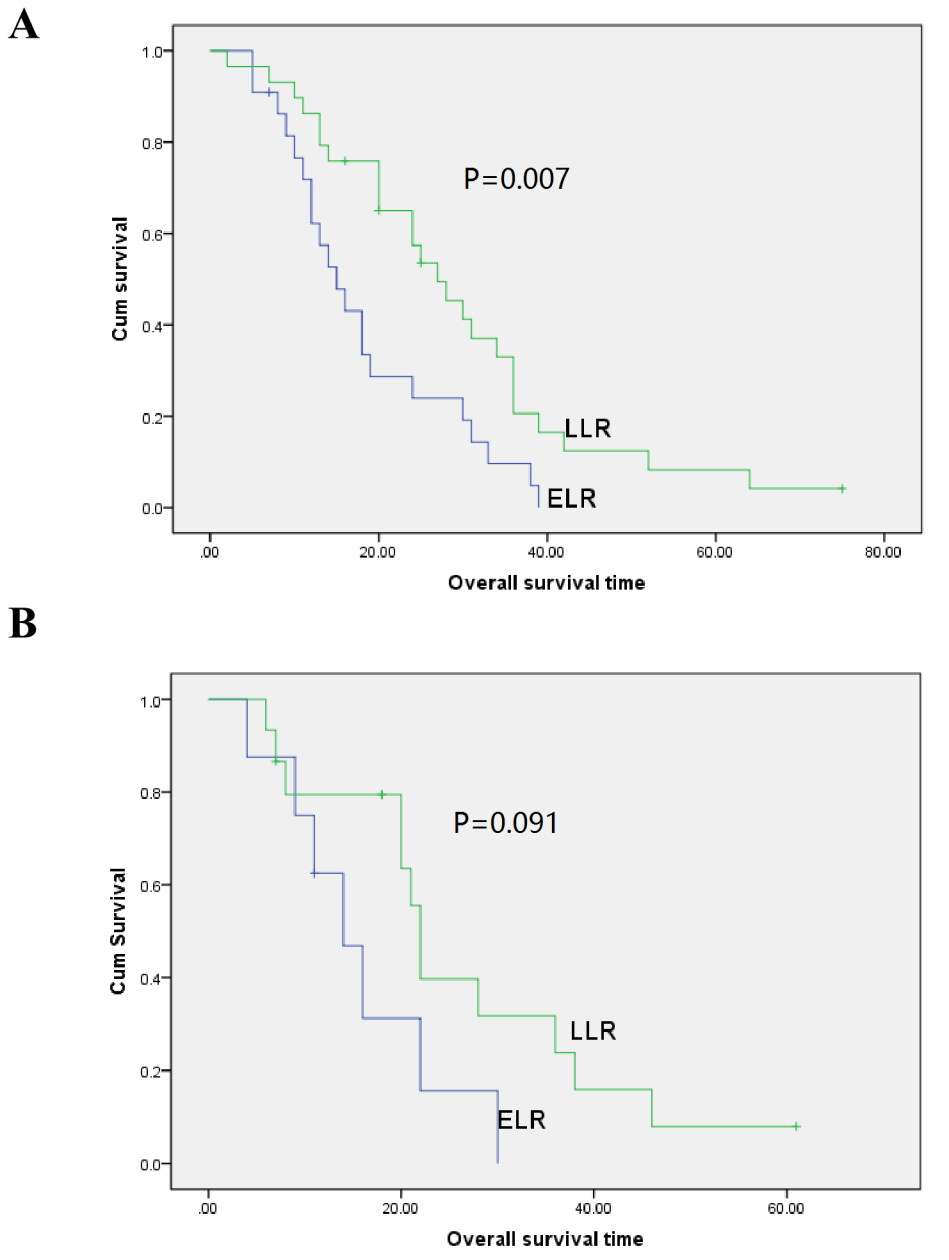
Overall survival curves with time to recurrence for gastric cancer patients with local recurrence in patients aged under (A., *P* = 0.007) and over (B., *P* = 0.091) 65 years

**Figure 3:**
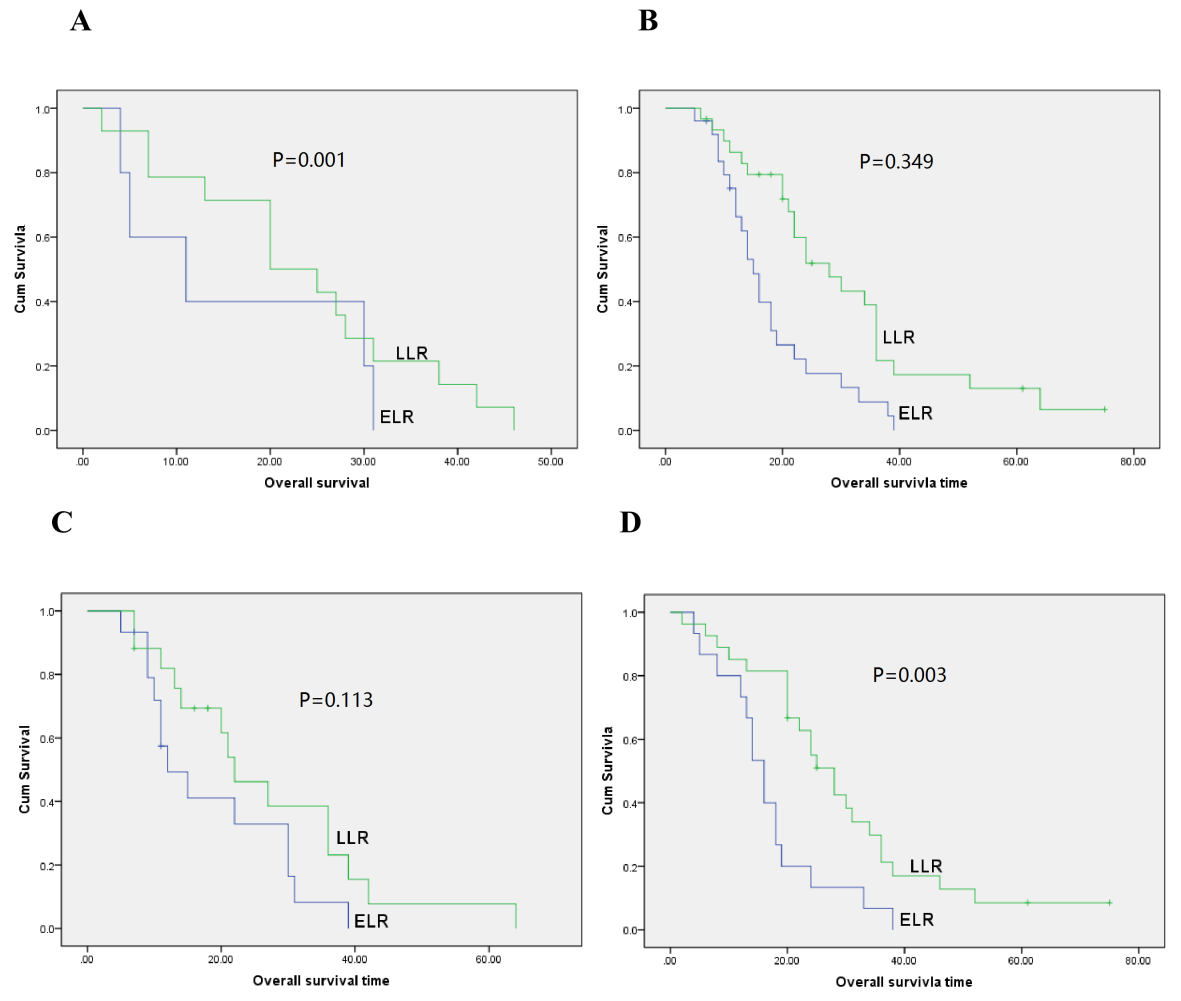
Overall survival curves with time to recurrence for gastric cancer patients with local recurrence in anastomotic recurrence patients (A., *P* = 0.001), local lymph node recurrence (B., *P* = 0.349), diameter of recurrent lesion < 2 cm (C., *P* = 0.113) and diameter of recurrent lesion ≥ 2 cm (D., *P* = 0.003)

**Figure 4:**
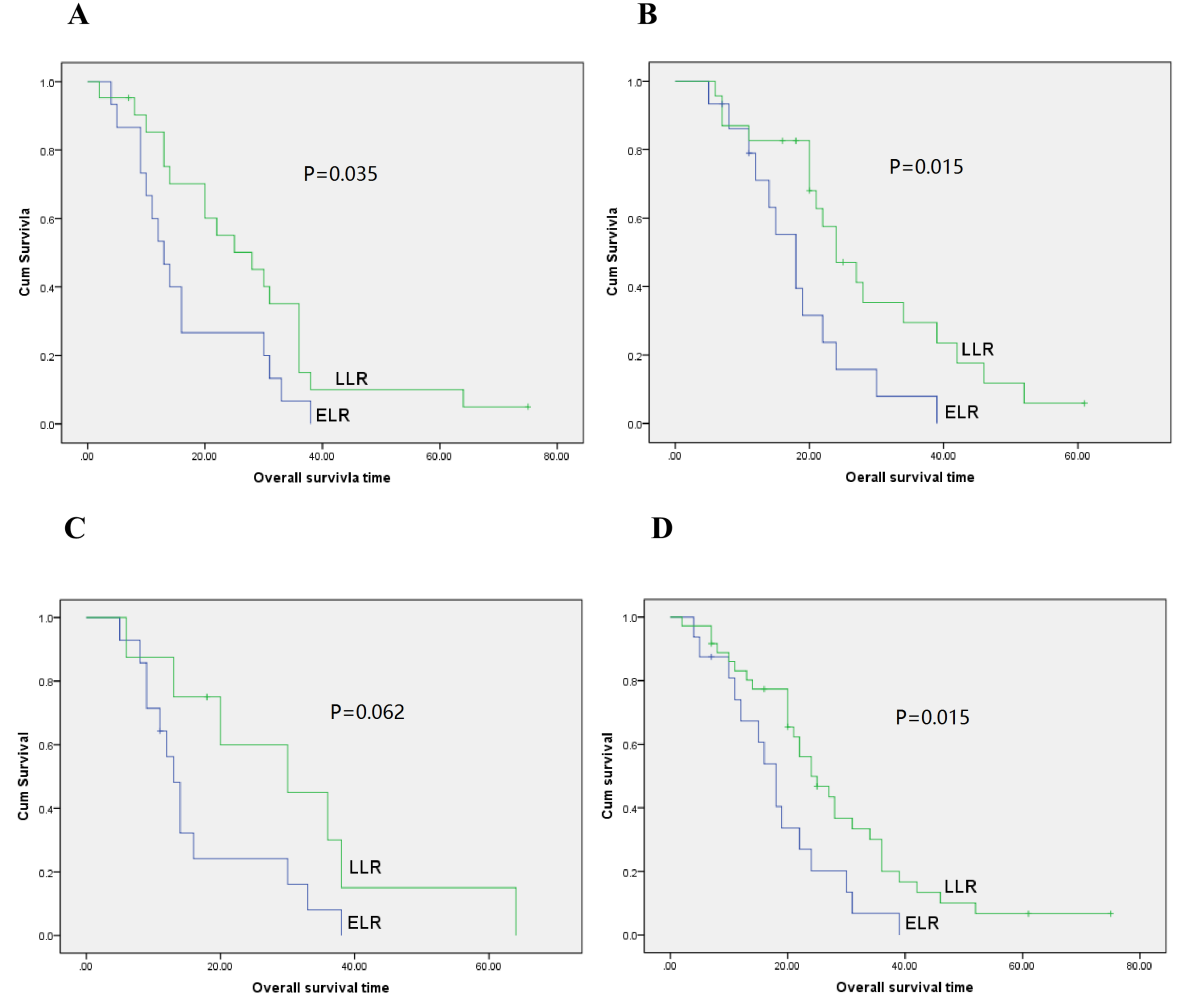
Overall survival curves with time to recurrence for gastric cancer patients with local recurrence in patients not receiving palliative chemotherapy (A., *P* = 0.035), receiving palliative chemotherapy (B., *P* = 0.015), not receiving radiosensitizing chemotherapy (C., *P* = 0.062) and receiving radiosensitizing chemotherapy (D., *P* = 0.015)

The overall follow-up duration after recurrence ranged from 2 to 75 months, with a median period of 20 months, during which 65 of 74 patients (87.8%) died due to the disease. The 1-, 2- and 3-year cumulative OS rates were 75.2%, 39.8% and 16.0%, respectively. Median OS was 15 months for ELR patients and 25 months for LLR patients. The 1-year and 2-year OS rates were 61.9%, 29.1% in ELR patients, but 76.9% and 51.6% for LLR patients, suggesting a significantly worse prognosis in ELR patients (*P* = 0.001, Figure 1A). Median OS time did not differ significantly between males in the ELR group and LLR group (*P* = 0.06, Figure 1B), however median OS time of female ELR patients was significantly shorter than that of female LLR patients (22 m *vs* 36 m, *P* = 0.002, Figure 1C). The 1-year OS rate was 23.9% and 54.7% in ELR patients and LLR patients aged under 65 years, respectively (*P* = 0.007, Figure 2A). However, in median OS did not differ significantly between ELR patients and LLR patients over the age of 65 (14 m *vs* 22 m, *P* = 0.091, Figure 2B). Interestingly, for patients with local lymph node recurrence, the 2-year OS rate was 17.7% for ELR patients and 51.9% for LLR patients, indicating worse prognosis in ELR patients (*P* = 0.001, Figure 3B). However, the 2-year OS rate did not differ significantly between anastomotic recurrence patients in the ELR and LLR group (*P* = 0.349, Figure 3A). Similarly, OS was shorter in the ELR than LLR group in patients with recurrence lesions with diameters of over than 2 cm (median OS: 16 m *vs* 28 m, *P* = 0.003, Figure 3D). But there was no difference in ELR than LLR group in patients with recurrence lesions with diameters of over than 2 cm (median OS: 12 m *vs* 22 m, *P* = 0.113, Figure 3C). OS also differed significantly between ELR and LLR patients with palliative chemotherapy (median OS: 13 m *vs* 28 m, *P* = 0.035, Figure 4A) and without palliative chemotherapy (median OS: 18 m *vs* 24 m, *P* = 0.015, Figure 4B).

We further analyzed the difference in OS between ELR and LLR patients according to application of radiosensitizing chemotherapy. Unexpectedly, in patients undergoing radiosensitizing chemotherapy prognosis was better for patients with LLR than those with ELR (median OS: 18 m *vs* 24 m, *P* = 0.015, Figure 4D). In patients that did not receive radiosensitizing chemotherapy, the median OS differed, but not significantly, between the ELR and LLR group (median OS: 13 m *vs* 30 m, *P* = 0.062, Figure 4C). We then used univariate analysis to analyze the factors which impact survival of patients with local recurrent gastric cancer (Table 3). We found that time to recurrence after primary surgery was significantly associated with survival (*P* = 0.002). Gender, treatment response and serum AFP level were not significantly associated with survival (gender: *P* = 0.106, treatment response: *P* = 0.093, serum AFP level: *P* = 0.211). The hazard ration (HR) of LLR compared with ELR was 0.442 (95% confidence interval: 0.264-0.741), suggesting that LLR was associated with a two-fold reduced risk of cancer-related death.

**Table 3:** Univariable analysis for the effect of time to local recurrence on overall survival after locoregional recurrence.

**Variable**	**Univariable analysis**		
	**HR**	**95%CI**	**P**
**Age**			
(<65 vs ≥65)	1.075	0.628-1.840	0.792
**Gender**			
(Male vs Female)	0.633	0.364-1.102	0.106
**T stage**			
(T1-2 vs T3-4)	0.876	0.445-1.726	0.703
**N stage**			
(N0-1 vs N2-3)	1.238	0.693-2.211	0.472
**Diameter of recurrent lesion**			
(<2 vs ≥2)	0.966	0.585-1.597	0.894
**Site of recurrence**			
Anastomotic recurrence vs	0.817	0.476-1.402	0.462
Local lymph node recurrence			
**Time between gastrectomy And recurrence**			
(<12m vs ≥12m)	0.442	0.264-0.741	0.002
**Palliative chemotherapy**			
(NO vs YES)	0.886	0.540-1.455	0.633
**Radiosensitizing Chemotherapy**			
(NO vs YES)	0.799	0.470-1.360	0.409
**Response**			
(CR+PR vs SD+PD)	1.610	0.923-2.807	0.093
**Serum CA 72-4**			
(≤6.9U/mL vs >6.9U/mL)	1.165	0.601-2.259	0.651
**Serum AFP**			
(≤25μg/L vs >25μg/L)	1.937	0.687-5.458	0.211
**Serum CEA**			
(≤5.9μg/L vs >5.9μg/L)	0.987	0.551-1.754	0.953
**Serum CA199**			
(≤37U/mL vs >37U/mL)	0.959	0.475-1.306	0.876

Next, we performed multivariate analysis to investigate which of these factors influenced prognosis (Table 4). Time to local recurrence was the only factor found to be significantly associated with OS time after local recurrence. ELR patients had almost a 2-fold increased risk of death than LLR patients (*P* = 0.012). These results suggest that time to recurrence predicts prognosis in unresectable gastric cancer with local recurrence.

**Table 4:** Multivariable analysis for the effect of time to local recurrence on overall survival after locoregional recurrence.

**Variables**	**Multivariable analysis**		
	**HR**	**95%CI**	**P**
**Gender**			
(Male vs Female)	0.675	0.386-1.182	0.169
**Time between gastrectomy And recurrence**			
(<12m vs ≥12m)	0.495	0.285-0.859	0.012
**Response**			
(CR+PR vs SD+PD)	1.268	0.69-2.324	0.443
**Serum AFP**			
(≤25μg/L vs >25μg/L)	2.095	0.738-5.943	0.165

## DISCUSSION

In this study, we found the mortality of gastric cancer patients with early local recurrence and no chance for further surgery to be higher mortality after diagnosis of local recurrence. Risk of death was almost two-fold higher in patients with early local recurrence than those with late local recurrence. We further demonstrated that the treatment response after radiotherapy with or without chemotherapy was associated with time to recurrence. These results suggest that early local recurrence indicates worse prognosis in unresectable gastric cancer patients with local recurrence.

Historically, local recurrence of gastric cancer was common [10]. Over recent years, rates of recurrence have been reduced by application of more extensive surgery and preoperative or postoperative chemoradiotherapy (CRT) [12]. However, local recurrence, which indicated high morbidity and mortality, is still frequently encountered in the clinic. Factors that affect survival after local recurrence have been described for other cancers, such as breast cancer and rectal cancer [13]. And these factors must be taken into account when making clinical decisions as a multidisciplinary team. Age and localization of recurrence have been reported to be prognostic factors in rectal cancer with local recurrence. Time to local recurrence is associated with overall survival time after diagnosis of ipsilateral recurrence in breast cancers [13]. Time to recurrence also has also been studies in rectal cancers [14], but the prognostic role of early local recurrence remains controversial [15]. The best treatment choice for local recurrence of gastric cancer is surgical resection. However, many patients are unsuitable for surgery. Thus, radiotherapy, chemotherapy or concurrent radiochemotherapy were applied. To our knowledge, the prognostic role of the time to recurrence in unresectable recurrent gastric cancer was not previously reported.

In this study ELR was defined as local recurrence diagnosed within 12 months of primary surgery and LLR was defined as local recurrence over 12 months after primary surgery, according to previous studies in other cancers [26]. We demonstrated that early local recurrence indicated worse prognosis in unresectable gastric cancer with local recurrence. Univariate and multivariate analysis indicated that time to recurrence was an independent prognostic factor for OS in local recurrent gastric cancers. Interestingly, the ORR of the therapy after recurrence in LLR group was 49.1% compared to19.0% in ELR group, and P value had significant difference. This result suggested time to recurrence was strongly associated with the ORR after therapy, indicating that patients with LLR may benefit more from the therapy. In other words, the treatments of the ELR patients need further investigation.

Until now, a few of studies focused on the prognostic factors associated the local recurrence of gastric cancer. Mehmedagic *et al* [27] found histological type and TNM stage of tumor were prognostic factors of local tumor recurrence of gastric adenocarcinoma. However, the type of surgery had no statistically significant value for tumor recurrence [16]. Antonio et al. found extension into the gastric wall thickness, spread to locoregional lymph nodes and the ability to generate distant metastases, as described by the TNM classification were closely related to the prognosis of patients [4]. We also analyzed the clinicopathological characteristics of primary tumors associated with recurrence. Interestingly, we found that stage and pathological types of primary tumors, and patient age and gender were not significantly associated with time to recurrence. These findings indicate that the aggressiveness of recurrent lesion growth differs from that of primary tumors. We also studied the relationship between time to recurrence and clinicopathological characteristics at time of locoregional recurrence. Interestingly, treatment response was associated with time to recurrence but not size or site of recurrent tumors. Serum biomarkers (AFP, CA72-4 and CA199), age and gender of patients were not related to time to recurrence. These results suggest that time to recurrence was associated with treatment response in unresectable gastric cancer with local recurrence.

Our conclusions are limited by the scope of this relatively small retrospective study in which local recurrence was detected *via* self-reported symptoms. Symptomatic patients may have a more extensive tumor growth than asymptomatic patients. Larger prospective studies will be required to assess the prognostic value of time to recurrence for unresectable gastric cancer with local recurrence.

In conclusion, the time between gastrectomy and recurrence is significantly associated with treatment response of unresectable gastric cancer with local recurrence. Time to recurrence may serve as an independent prognostic factor for overall survival after local recurrence in unresectable gastric cancers. All patients with local recurrence who were unsuitable candidates for further surgery should assess the time to recurrence to predict prognosis of patients.

## MATERIALS AND METHODS

We retrospectively reviewed the medical records of 74 gastric cancer patients with local recurrence after radical gastrectomy. For all patients, radical surgery (R0 resction, D2 lymphadebectomy) was operated for the primary tumors for I-III stage gastric cacner. Adjuvant chemotherapy with Capecitabine plus Oxaliplatin or S-1 was started within 6 weeks after surgery and continued for 6 or 12 months for stage II-III patients and stage I patients with high risk factors (such as poor differentiation, signet ring cell carcinoma, vessel carcinoma embolus and age < 50years old ). Patients were excluded if they had other malignancies or distant metastasis. Local recurrence was detected *via* follow-up screening or self-reported symptoms. Every patient who was diagnosed as locoregional recurrence was evaluated using the CT scan to determine the clinical stage. Anastomotic recurrence and nodal recurrence in the stations 1-11 lymph nodes and stations 12-16 lymph nodes recurrence which included posterior pancreatic lymph nodes, mesenteric lymph nodes, middle colic lymph nodes, para-aortic lymph nodes and the lymph nodes in the hepatic duodenal ligament were defined as local recurrence. For the patients with stations 12-16 lymph nodes recurrence, the recurrent lesions were include in in-field radiotherapy area that evaluated by a radiation oncologist in our hospital. All patients with gastric cancer were evaluated by the surgeons in Multidisciplinary treatment (MDT). And no patients were suitable candidates for additional surgery; thus these patients were defined as unresectable locoregional recurrence. A diagnostic radiologist reviewed the imaging and measured the diameter of recurrent lesion using the imaging studies. As for the synchronously anastomotic and lymph node recurrence, the diameter of current lesion was defined as the diameter of the biggest recurrent lesion. After local recurrence, all patients received field radiotherapy with or without palliative chemotherapy from January 1997 to December 2014 at Zhejiang Cancer Hospital. All patients received field radiotherapy (44.0 Gy-55.0 Gy) with or without radiosensitizing chemotherapy. The radiosensitizing chemotherapy consisted of S1 at 50 mg/m^2^ or Gemicitabine at 1.0 g/m^2^ twice daily from days 1 to 14 during the radiotherapy. And palliative chemotherapy consisted of taxane-based regimen (paclitaxel or docetaxel plus S-1/gemicitbine/5-fluorouracil), oxaliplatin-based regimen (oxaliplatin or oxaliplatin plus S-1/gemcitabine/5-fluorouracil/paclitaxel) and irinotecan-based regimen (irinotecan or irinotecan plus S-1/5-fluorouracil). The clinicopathological characteristics of primary and recurrent tumors were retrospectively collected and analyzed for each patient: age, sex, TNM stage (using the AJCC TNM staging system), situation of vascular invasion, pathological type, size of recurrent lesion, site of recurrence, treatment response and OS after recurrence. The patients were divided into two groups according to time to local recurrence. Early local recurrence was defined as recurrence diagnosed less than 12 months after primary surgery. And a local recurrence diagnosed 12 or more months after primary surgery was defined as a late local recurrence. Overall survival was calculated as the time from diagnosis of local recurrence to death or censoring. This study protocol was approved by the institutional review board of our hospital.

### Statistical analysis

The chi-square test was performed to assess the relationship between clinicopathological variables and time to recurrence. Survival was estimated using the Kaplan-Meier method. The significance of differences between curves was calculated using the log-rank test. The Cox proportional hazards model with the backward selection method was performed for multivariate analysis. All statistical calculations were performed with SPSS 13.0 for Windows (Chicago, IL). Two-sided *P* values of < 0.05 were considered to represent statistical significance.
